# Accelerated spin dynamics using deep learning corrections

**DOI:** 10.1038/s41598-020-70558-1

**Published:** 2020-08-13

**Authors:** Sojeong Park, Wooseop Kwak, Hwee Kuan Lee

**Affiliations:** 1 Department of Physics, Chosun University, Gwangju, 61452 Republic of Korea; 2 Bioinformatics Institute, Agency for Science, Technology and Research (A*STAR), 30 Biopolis Street, #07-01 Matrix, Singapore, 138671 Singapore; 3 School of Computing, National University of Singapore, 13 Computing Drive, Singapore, 117417 Singapore; 4 Singapore Eye Research Institute (SERI), 11 Third Hospital Ave, Singapore, 168751 Singapore; 5 Image and Pervasive Access Laboratory (IPAL), 1 Fusionopolis Way, #21-01 Connexis (South Tower), Singapore, 138632 Singapore

**Keywords:** Phase transitions and critical phenomena, Statistical physics, Magnetic properties and materials, Phase transitions and critical phenomena, Computational science

## Abstract

Theoretical models capture very precisely the behaviour of magnetic materials at the microscopic level. This makes computer simulations of magnetic materials, such as spin dynamics simulations, accurately mimic experimental results. New approaches to efficient spin dynamics simulations are limited by integration time step barrier to solving the equations-of-motions of many-body problems. Using a short time step leads to an accurate but inefficient simulation regime whereas using a large time step leads to accumulation of numerical errors that render the whole simulation useless. In this paper, we use a Deep Learning method to compute the numerical errors of each large time step and use these computed errors to make corrections to achieve higher accuracy in our spin dynamics. We validate our method on the 3D Ferromagnetic Heisenberg cubic lattice over a range of temperatures. Here we show that the Deep Learning method can accelerate the simulation speed by 10 times while maintaining simulation accuracy and overcome the limitations of requiring small time steps in spin dynamic simulations.

## Introduction

Magnetic materials have a wide range of industrial applications such as in Nd–Fe–B-type permanent magnets used for motors in hybrid cars^1,2^, magnetoresistive random access memory (MRAM) based on the storage of data in stable magnetic states^3^, ultrafast spins dynamics in magnetic nanostructures^4,5^, heat assisted magnetic recording and ferromagnetic resonance methods for increasing the storage density of hard disk drives^6,7^, exchange bias related to magnetic recording^8^, and magnetocaloric materials for refrigeration technologies^1^. Understanding the underlying physics of magnetic material enables us to develop much better applications. In particular, the study of the properties of these magnetic materials is performed experimentally by using neutron scattering^9^. Magnetic properties of materials are also studied theoretically using computational methods. Spin dynamics simulations^10^ are powerful tools for understanding fundamental properties of magnetic materials that can be verified by experimental methods. In spin dynamics simulations, classical equations of motion of spin systems are solved numerically using well known integrators such as leapfrog, Verlet, predictor-corrector, and Runge-Kutta methods^11–13^. The accuracy of these simulations depends on a time integration step size. If a large time step is used, the accumulated truncation error becomes larger. Conversely, using a short time step is very computationally demanding. So, it is important to find a trade off between speed and accuracy.

Symplectic methods^14,15^ are among the most useful time integrators for spin dynamics simulations. The numerical solutions of symplectic methods have properties of the time reversibility and the energy conservation. For example, high order Suzuki–Trotter decomposition method, one of the symplectic methods, allows for larger time step with limited error in its computation. In this paper, we seek to enhance the time integration step of Suzuki–Trotter decomposition method further using Deep Learning techniques. For second-order Suzuki–Trotter decomposition method, the integration time step is limited up to $~\uptau \sim 0.04/J$ and for fourth-order Suzuki–Trotter decomposition method, the integration time step is limited up to $~\uptau \sim 0.2/J$^16^.

Recently, Machine Learning techniques are used to enhance simulation efficiencies in the condensed matter physics. Its applications include addressing difficulties of phase transition^17–22^ and accelerating the Monte-Carlo simulations^23^. A crucial issue in molecular dynamics simulations^24^ is that generating samples from the equilibrium distributions is time consuming. Boltzmann generators machine^25^ addresses the long-standing rare-event (e.g. transition) sampling problem.  In addition, study of quantum many body systems using Machine Learning is applied to simulation of the quantum spin dynamics^26,27^, identifying phase transitions^28^, and solves the exponential complexity of the many body problem in quantum systems^29^.

In this paper, we show that speed up is achieved if we combine spin dynamics simulation and Deep Learning to learn the error corrections. The first condition for speed up is enough capacity of Deep Learning to learn the associations between spin configuration generated by large time steps and spin configuration generated by accurate short time steps. The second condition is enough training data for learning and show the Deep Learning enough pairs of patterns between spin configuration for large and short time steps. We propose to use Deep Learning to estimate the error correction terms of Suzuki–Trotter decomposition method, and then add the correction terms back to spin dynamics results, making them more accurate. As a result of this correction, larger time step can be used for Suzuki–Trotter decomposition method, and corrections can be made for each time step. To evaluate our Deep Learning method, we analyze spin-spin correlation as a more stringent measure. We also use thermal averages to benchmark the performance of our method. We compare the Deep Learning results with those from spin dynamics simulation without Deep Learning for short time steps.

## Methods

**Figure 1:**
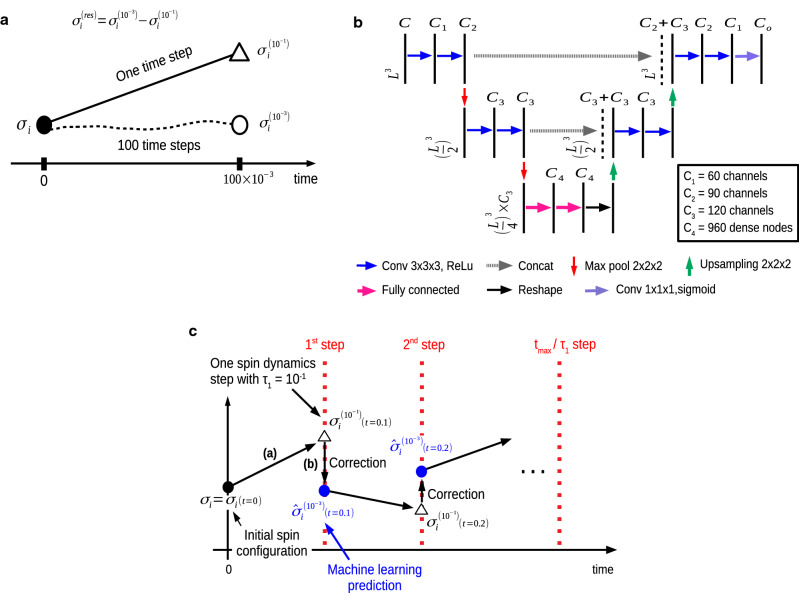
Deep learning for Heisenberg model. **a** Spin configurations for training data preparation. $\sigma _i$ is initial spin configuration, $\sigma _{i}^{(10^{-1})}$ is spin configuration after one time step of ${\uptau _{1}=10^{-1}}$ from $\sigma _i$, and $\sigma _{i}^{(10^{-3})}$ is spin configuration after 100 time steps of ${\uptau _{3}=10^{-3}}$ from $\sigma _i$. $\sigma _{i}^{(res)}$ is residue of $\sigma _{i}^{(10^{-3})}$ and $\sigma _{i}^{(10^{-1})}$. **b** Illustration of the U-Net architecture. Each vertical black line represents a multi-channel feature map. The number of channels is denoted on the top of the straight vertical black line and each map’s dimension is indicated on the left edge. Vertical dashed black lines correspond on the copied feature maps from each encoder layer. **c**, A sequence of spin dynamics for testing the trained U-Net model: (a) conduct one time step $\uptau _{1} = 10^{-1}$ of spin dynamics simulation; (b) use $\sigma _{i}^{(10^{-1})}$ to predict the spin configuration ${\sigma }_i^{(10^{-3})}$ by estimating predicted residue ${\hat{\sigma }}^{(res)}_i$ using Eq. (6); Steps (a) and (b) are repeated up to t$_{max}$ time.

**Figure 2:**
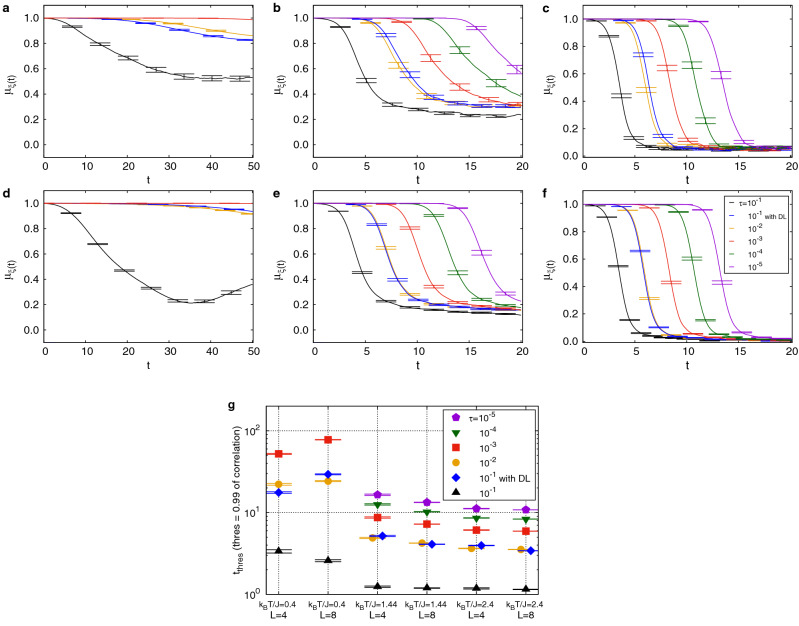
Spin-spin correlation using reference trajectory generated at ${\uptau =10^{-6}}$. Analysis of the mean of correlation $\mu _{\xi (t)}$ as a function of time on $4 \times 4 \times 4$ cubic lattice at $\mathbf{a} , k_{B}T/J=0.4$, $\mathbf{b} , k_{B}T_{c}/J\approx 1.44$, and $\mathbf{c} , k_{B}T/J=2.4$ and those on $8 \times 8 \times 8$ cubic lattice at $\mathbf{d} , k_{B}T/J=0.4$, $\mathbf{e} , k_{B}T_{c}/J\approx 1.44$, and $\mathbf{f} , k_{B}T/J=2.4$. Blue line presents the Deep Learning (DL) result while black line, yellow line, and red line are the simulation results for $\uptau =10^{-1}$, $\uptau =10^{-2}$, and $\uptau =10^{-3}$, respectively. Especially, at $k_{B}T/J=1.44$ and $k_{B}T/J=2.4$, green line and violet line show the simulation results for $\uptau =10^{-4}$ and $\uptau =10^{-5}$, respectively. **g** Threshold time $t_{thres}$ as function of temperature. Filled rhombi ( ) represents the Deep Learning result while filled black triangles($\blacktriangle$), filled yellow circles ( ), filled red squares ( ), filled green inverted triangles ( ), and filled violet pentagons ( ) are the simulation results without DL corrections for $\uptau =10^{-1}$, $\uptau =10^{-2}$, $\uptau =10^{-3}$, $\uptau =10^{-4}$, and $\uptau =10^{-5}$, respectively.

### Heisenberg model

The ferromagnetic Heisenberg model on a cubic lattice is used to demonstrate the efficiency of our method. The Hamiltonian for this model is given as ${H} = -J\sum _{<i,j>}\mathcal {{\varvec{S}}}^i \cdot \mathcal {{\varvec{S}}}^j$, where a vector ${\varvec{S}}^i$ has three components $(S^{i}_x,S^{i}_y,S^{i}_z)$ and $|{\varvec{S}}^i|$ is a unit vector. We formalize our spin dynamics following the notations of Tsai ${et \ al}.$^16^. We write the equations of motion for all spins as

1 $$\begin{aligned} \begin{aligned} \frac{d \sigma (t)}{d t} = {\hat{R}} \sigma (t) , \end{aligned} \end{aligned}$$

where $\sigma (t)=({\varvec{S}}^1(t),{\varvec{S}}^2(t),\dotsc ,{\varvec{S}}^n(t))$ is the spin configuration at time t. The integration of the equations of motion in Eq. (1) is done using the second order Suzuki–Trotter decomposition method as in Tsai ${et \ al.}$^16^. As following the mathematical notations of Tsai et al., we decompose the evolution operator $\hat{R}$ into $\hat{R}_A$ and $\hat{R}_B$ on the sublattices A and B respectively, and obtain

2 $$\begin{aligned} \begin{aligned} {\text { e}}^{({\hat{R}}_A + {\hat{R}}_B) \uptau } = {\text { e}}^{{\hat{R}}_B \uptau /2}{\text {e}}^{{\hat{R}}_A \uptau }{\text { e}}^{{\hat{R}}_B \uptau /2} + O(\uptau ^3) \end{aligned} \end{aligned}$$

The ferromagnetic Heisenberg model is considered on the cubic lattice of dimensions $L\times L \times L$ with periodic boundary conditions. This model undergoes a phase transition at a temperature ${k_B}T_{c}/J=1.442 \dots$^30^, where $k_B$ is Boltzmann’s constant. In the spin dynamics approach, the equations of motion for the Heisenberg model is governed by the following equation:

3 $$\begin{aligned} \begin{aligned} \frac{d{\varvec{S}}^i}{dt}=\mathcal {-{\varvec{S}}}^i \times \mathcal {{\varvec{H}}}_{ \text{ eff }}^i= \begin{bmatrix} 0 &\quad{} -H_{ \text{ eff,z }}^{i} &\quad{} H_{ \text{ eff,y }}^{i} \\ H_{ \text{ eff,z }}^{i} &\quad{} 0 &\quad{} -H_{ \text{ eff,x }}^{i} \\ -H_{ \text{ eff,y }}^{i} &\quad{} H_{ \text{ eff,x }}^{i} &\quad{} 0 \end{bmatrix}\mathcal {{\varvec{S}}}^i =R^i \mathcal {{\varvec{S}}}^i. \end{aligned} \end{aligned}$$

Here, $\mathcal {{\varvec{H}}}_{ \text{ eff }}^i$ is the effective field acting on the *i*th spin. The *k* component of the effective field can be specified as ${{\varvec{H}}}^{i}_{ \text{ eff,k }}$ = $-\sum _{j=nn(i)}{S}^{j}_k$, where the sum runs over the nearest neighbor pairs of sites and $k=x,y,$ and *z*.

**Figure 3:**
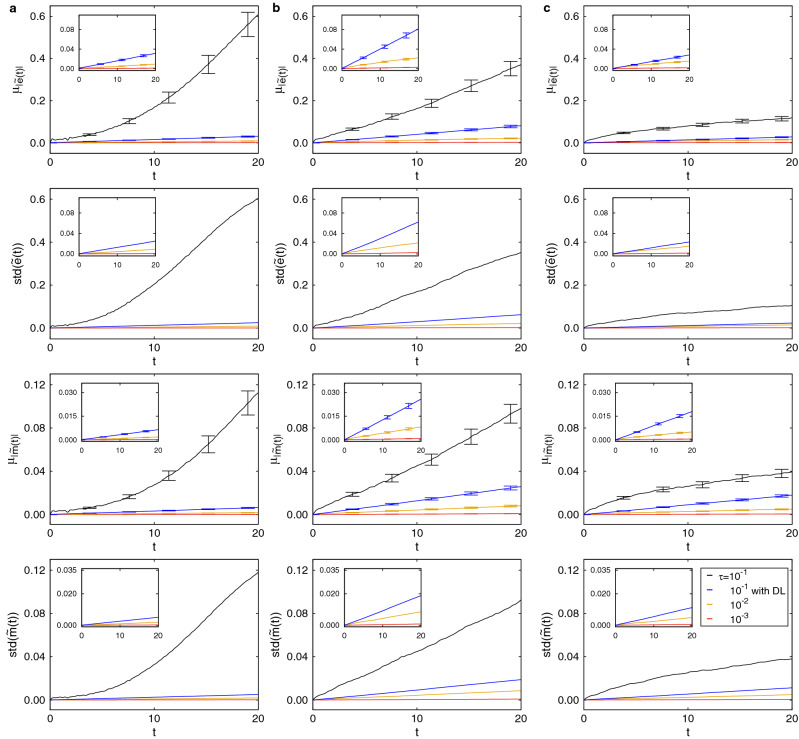
Conservation of energy and magnetization on ${4 \times 4 \times 4}$ cubic lattice. Predictions of the mean of absolute energy per site $\mu _{|{\tilde{e}}(t)|}$, standard deviation of energy per site std$({\tilde{e}}(t))$, the mean of absolute magnetization per site $\mu _{|{\tilde{m}}(t)|}$, and standard deviation of magnetization per site std$({\tilde{m}}(t))$ as a function of time at $\mathbf{a }, k_{B}T/J=0.4$, $\mathbf{b }, k_{B}T_{c}/J\approx 1.44$, and $\mathbf{c }, k_{B}T/J=2.4$. Black line, yellow line, and red line represent data obtained from spin dynamics simulations with $\uptau =10^{-1}$, $\uptau =10^{-2}$, and $\uptau =10^{-3}$, respectively, while blue line represents data from Deep Learning (DL) correction.

**Figure 4:**
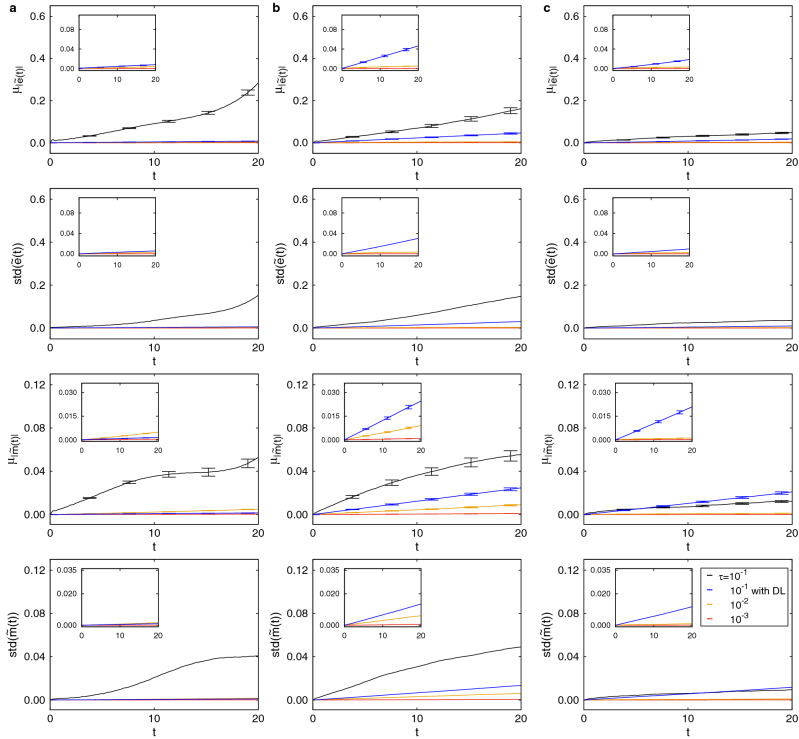
Conservation of energy and magnetization on ${8 \times 8 \times 8}$ cubic lattice. Predictions of $\mu _{|{\tilde{e}}(t)|}$, std$({\tilde{e}}(t))$, $\mu _{|{\tilde{m}}(t)|}$, and std$({\tilde{m}}(t))$ as a function of time at $\mathbf{a }, k_{B}T/J=0.4$, $\mathbf{b }, k_{B}T_{c}/J\approx 1.44$, and $\mathbf{c }, k_{B}T/J=2.4$. Black line, yellow line, and red line represent data obtained from spin dynamics simulations with $\uptau =10^{-1}$, $\uptau =10^{-2}$, and $\uptau =10^{-3}$, respectively, while blue line represents data from Deep Learning (DL) correction. These figures show that the effect of averaging over disordered spins for $L=8$ is stronger than for $L=4$ above the critical temperature ${k_B}T_{c}/J$.

### Deep Learning approach

A fully supervised Deep Learning method is developed to perform the spin dynamics by using the second order Suzuki–Trotter decomposition method to reduce simulation errors. In order to produce training data for our supervised Deep Learning, initial spin configurations are considered at ordered, near-critical, and disordered states in the temperature range ${{k_B}T/J} \in [0.5,2.4]$ and sampling ${9.1 \times 10^5}$ independent spin configurations using Monte-Carlo simulations with the Metropolis–Hastings algorithm^30–33^. The initial spin configurations are prepared with 300,000 samples in ordered states, 210,000 samples near critical states, and 400,000 samples disordered states by simulated annealing method. The temperature annealing scheme will be described in more details in the supplementary information. The temperatures for annealing are gradually lowered from high to low temperatures and Monte Carlo data are always obtained at equilibrium configurations. For each sampled initial spin configuration $\sigma _{i}$, two sets of spin dynamics simulations are performed with the time steps ${\uptau _{1}=10^{-1}}$ and ${\uptau _{3}=10^{-3}}$ as illustrated in Fig. 1a. Second-order Suzuki–Trotter method uses ${\uptau =0.04}$ as typical integration time step, so we use ${\uptau =10^{-3}}$ which would give good accurate simulation. For large time step, we tried ${\uptau =10^{-2}}$ and ${\uptau =10^{-1}}$, with our Deep Learning corrections, a large time step of ${\uptau =10^{-1}}$ gives the best speed up with a good accuracy. The spin configuration with time step ${\uptau _{3}=10^{-3}}$ needs 100 time steps of simulations to pair with the spin configuration with one time step ${\uptau _{1}=10^{-1}}$. Formally, we represent the updated spin configurations $\sigma _{i}^{(10^{-1})}$ and $\sigma _{i}^{(10^{-3})}$ by using the Suzuki–Trotter decomposition method as

4 $$\begin{array}{*{20}l} {\sigma _{i}^{{(10^{{ - 1}} )}} \leftarrow {\text{e}}^{{\hat{R}_{B} \tau _{1} /2}} {\text{e}}^{{\hat{R}_{A} \tau _{1} }} {\text{e}}^{{\hat{R}_{B} \tau _{1} /2}} \sigma _{i} ,} \hfill & {\tau _{1} = 10^{{ - 1}} } \hfill \\ {\sigma _{i}^{{(10^{{ - 3}} )}} \leftarrow ({\text{e}}^{{\hat{R}_{B} \tau _{3} /2}} {\text{e}}^{{\hat{R}_{A} \tau _{3} }} {\text{e}}^{{\hat{R}_{B} \tau _{3} /2}} )^{{100}} \sigma _{i} ,} \hfill & {\tau _{3} = 10^{{ - 3}} \quad i = 1, \ldots D,} \hfill \\ \end{array}$$

where ${\sigma }_i$ is an initial spin configuration and *D* represents the number of training data. The difference between spin configuration $\sigma _{i}^{(10^{-3})}$ generated using ${\uptau _{3}=10^{-3}}$ and spin configuration $\sigma _{i}^{(10^{-1})}$ generated using ${\uptau _{1}=10^{-1}}$ is captured by

5 $$\begin{aligned} \sigma _{i}^{(res)} = \sigma _{i}^{(10^{-3})}-\sigma _{i}^{(10^{-1})} \quad \quad \quad \ i=1, \dots ,D \ , \end{aligned}$$

where $\sigma _{i}^{(res)}$ is residue. For our Deep Learning, initial spin configuration $\sigma _{i}$ and spin configuration $\sigma _{i}^{(10^{-1})}$ are used as the inputs into U-Net^34^, a kind of convolutional neural networks. The U-Net is a proven architecture for image segmentation as well as for extracting subtle features. The detailed structure of U-Net is shown in Fig. 1b. The architecture of U-net used for $8 \times 8 \times 8$ cubic lattice is that convolutional layers are used as an encoder on left upper side followed by a decoder on right upper side that consists of upsamplings and concatenations with the correspondingly feature maps from the encoder. We add fully connected layers (FC) in the bottom of the network between the encoder and the decoder to efficiently determine particular weights in the feature map from the encoder , such as capturing more information of spin-spin interactions. The input channels *C* are 6 by concatenating spin coordinates $S_x$, $S_y$, and $S_z$ of both $\sigma _{i}$ and $\sigma _{i}^{(10^{-1})}$, respectively. The input dimensions of U-Net are reshaped to [*D*, *L*, *L*, *L*, *C*] as cubic grid vector map, where *D* is the total number of training data, *L* is lattice size, and *C* is input channels. The encoder consists of the repeated two convolutional layers with $3 \times 3 \times 3$ filters followed by a $2 \times 2 \times 2$ max pooling. We apply a reshaping function to FC with dimensions from $[D,\frac{L}{4} \times \frac{L}{4} \times \frac{L}{4} \times C_4]$ into $[D,\frac{L}{4},\frac{L}{4},\frac{L}{4},C_4]$. Every step in decoder consists of upsampling layers with a $2 \times 2 \times 2$ filters followed by the repeated two convolutional layers with $3 \times 3 \times 3$ filters and copies with correspondingly cropped feature maps from encoding layers. The periodic boundary conditions are also applied to the convolutional layers. The activation function of the output is a sigmoid for predicting values of residue with $[D,L,L,L,C_o]$ dimensions, where the number of output channels $C_o$ is 3. A simpler U-Net architecture is used for $4 \times 4 \times 4$ cubic lattice (see supplementary information).

### Deployment of our U-Net for spin dynamics

To deploy the trained U-Net for spin dynamics, spin dynamics simulation is carried out with one large time step $\uptau _{1} = 10^{-1}$ and this simulation result $\sigma _{i}^{(10^{-1})}$ can be used to predict $\sigma _{i}^{(10^{-3})}$ as follows:

6 $$\begin{aligned} {{\hat{\sigma }}_{i}^{(10^{-3})}} = {\sigma _{i}^{(10^{-1})} + {\hat{\sigma }}^{(res)}_i } \simeq {\sigma }_{i}^{(10^{-3})} , \end{aligned}$$

where ${\hat{\sigma }}_i^{(10^{-3})}$ is the predicted spin configuration for 100 time steps of $\uptau _{3} = 10^{-3}$ and predicted residue ${\hat{\sigma }}^{(res)}_i$ is the correction term by Deep Learning. A sequence of spin dynamics are conducted at $\uptau _{1} = 10^{-1}$ and for each step, Eq. (6) is used to perform corrections as shown in Fig. 1c. This new time integration scheme is repeated up to maximum time t$_{max}$. This scheme requires only forward propagation using the GPU implemented with TensorFlow library^35^, so the computing time is negligible.

### Normalization of residue

The difference between spin configuration generated with ${\uptau _{3}=10^{-3}}$ and that generated with ${\uptau _{1}=10^{-1}}$ is captured by residue $\sigma _{i}^{(res)}$ in Eq. (5). Let $(\sigma _{i}^{(res)})_k^j$ be the *k* component of residual spin at site *j* of the lattice, and *k* denotes *x*, *y*, and *z* components. The values of $(\sigma _{i}^{(res)})_k^j$ can be quite small for some simulations, to maintain numerical stability, we normalize these values as follows. Each component $(\sigma _{i}^{(res)})_k^j$ over *D* samples of training data is normalized to a range of [0,1] by fitting to have a Gaussian distribution, and find the mean and standard deviation for each *k* component, respectively.

For lattice size $L=4$, $\lambda _{min}=-0.22455$ and $\lambda _{max}=0.22455$ are defined by taking 11 times the largest standard deviation of *k* component. 11 standard deviations translates to a p-value of $1.911 \times 10^{-28}$, which ensures that during inference, the normalized residue $(\sigma _{i}^{(res)})_k^j$ is always within the range [0,1]. For lattice size $L=8$, $\lambda _{min}=-0.25472$ and $\lambda _{max}=0.25472$ are defined by taking 13 times the largest standard deviation of *k* component. Finally, each component $(\sigma _{i}^{(res)})_k^j$ is normalized to the range [0, 1] and guarantee stable convergence of weights and biases in Deep Learning as follows :

7 $$\begin{aligned} (\sigma _{i}^{norm})_k^j = \frac{(\sigma _{i}^{(res)})_k^j - \lambda _{min}}{\lambda _{max} - \lambda _{min}} \ \ \ \ (k=x,y,z, \ i=1,...D). \end{aligned}$$

During the prediction, $(\sigma _{i}^{(res)})_k^j$ from test data is normalized to a range of [0, 1] by using $\lambda _{min}$ and $\lambda _{max}$, which have already been obtained.

### Loss function and training

The loss function for one data point of $(\sigma _{i},\sigma _{i}^{(10^{-1})},\sigma _{i}^{norm})$ is the mean-square error between the normalized residue $\sigma _{i}^{norm}$ and the predicted normalized residue ${\hat{\sigma }}_{i}^{norm}$ and is defined as

8 $$\begin{aligned} \begin{aligned} {\mathcal {L}}(\sigma _{i},\sigma _{i}^{(10^{-1})},\sigma _{i}^{norm}) = \frac{1}{L^3}{\sum _{j=1}^{L^3}\left\Vert (\sigma _{i}^{norm})^j-({\hat{\sigma }}_{i}^{norm})^j\right\Vert _2^2} \ , \end{aligned} \end{aligned}$$

where *j* is the index of lattice sites. The distance function between the $j^{th}$ site of $\sigma _{i}^{norm}$ and the $j^{th}$ site of ${\hat{\sigma }}_{i}^{norm}$ is the sum of the square difference of all spin components :

9 $$\begin{aligned} \begin{aligned} \left\Vert (\sigma _{i}^{norm})^j-({\hat{\sigma }}_{i}^{norm})^j\right\Vert _2^2 = \sum _{k=x,y,z}\left( \left( \sigma _{i}^{norm} \right) _k^j-\left( {\hat{\sigma }}_{i}^{norm}\right) _k^j\right) ^2 \ , \end{aligned} \end{aligned}$$

where *i* is the index of training data.

### Converting ${{\hat{\sigma }}^{{norm}}_i}$ to ${{\hat{\sigma }}^{{(res)}}_i}$

For our Deep Learning, inputs into U-Net are obtained initial spin configurations $\sigma _{i}$ and spin configurations $\sigma _{i}^{(10^{-1})}$ generated by spin dynamics simulations, and output is ${\hat{\sigma }}_{i}^{norm}$. We finally predict the spin configuration for 100 time steps of $\uptau _{3} = 10^{-3}$ using trained Deep Learning model as ${{\hat{\sigma }}_{i}^{(10^{-3})}} = {\sigma _{i}^{(10^{-1})} + {\hat{\sigma }}^{(res)}_i }$, where the predicted residue ${\hat{\sigma }}^{(res)}_i$ can be obtained by the following converting formula as ${{\hat{\sigma }}^{(res)}_i = {\hat{\sigma }}_{i}^{norm}(\lambda _{max} - \lambda _{min}) + \lambda _{min} }$.

## Results

The effectiveness of our proposed Deep Learning method is evaluated at ${k_B}T/J=0.4<{k_B}T_{c}/J$, ${k_B}T/J=1.44 \approx {k_B}T_{c}/J,$ and ${k_B}T/J=2.4>{k_B}T_{c}/J$. Note that at ${k_B}T/J=2.4$, the system is in a disordered state and spatial corrections between spins are very short. One hundred independent spin configurations are generated by using Monte-Carlo simulation for use as test data sets at each temperature ${k_B}T/J=0.4$, 1.44, and 2.4. Second order Suzuki–Trotter decomposition methods are used for all experiments in this paper.

To evaluate the accuracy of simulation results, correlation is investigated by comparing spin dynamics trajectory $\sigma (t)$ with highly accurate spin dynamics trajectory $\rho (t)$ performed with $\uptau =10^{-6}$. $\uptau =10^{-6}$ is used as the reference time step as we found that it can give accurate trajectories. Correlation $\xi (t)$ as function of time *t* in which $\sigma (t)$ and $\rho (t)$ are compared is given by

10 $$\begin{aligned} \begin{aligned} {\xi (\sigma , t)}&= {\frac{1}{L^{3}}}{\sum _{j=1}^{L^3} [(\rho ^{j}(t))_{x} (\sigma ^{j}(t))_{x} + (\rho ^{j}(t))_{y} (\sigma ^{j}(t))_{y} + (\rho ^{j}(t))_{z} (\sigma ^{j}(t))_{z}]}, \end{aligned} \end{aligned}$$

where index *j* denotes lattice site of spins, *L* is the linear dimension of the lattice, and $L^3$ is total number of spins at lattice sites. Since the initial spin configurations are the same, $\rho (0)$ is identical to $\sigma (0)$. We compute one hundred correlation $\xi ({\sigma }_{i},t)$ for spin configurations ${\sigma }_{i}(t)$, where *i* is from 1 to 100. Then, we also estimate the mean of correlation $\mu _{\xi (t)}$ and the standard deviation of correlation std$\left( {\xi (t)}\right)$ of $\xi ({\sigma }_{i},t)$ as a function of time at each temperature.

Suzuki–Trotter decomposition method provides important properties such as conservation of energy ${{\varvec{e}}}=-{L^{-3}}\sum _{<i,j>}^{L^3}\mathcal {{\varvec{S}}}^i \cdot \mathcal {{\varvec{S}}}^j$ and magnetization ${{\varvec{m}}} = {L^{-3}}{ \sqrt{\left( \sum _i S^{i}_x\right) ^{2}+\left( \sum _i S^{i}_y\right) ^{2}+\left( \sum _i S^{i}_z\right) ^{2}}}$ , and time reversibility. We wish to compare the conservation of energy and magnetization across one hundred samples, but their starting spin configurations are different. In order to take statistics across the samples, we shift the energy and magnetization of the initial spin configurations to zero. Eq. (11) and Eq. (12) show how we shift the energy per site *e*(*t*) and magnetization per site *m*(*t*) at each time step *t*. Here, Q represents the number of samples at each temperature. We use Q as one hundred.

11 $$\begin{aligned} {{\tilde{e}}_i}(t)&= {} e_{i}(t)- e_{i}(0) \quad i=1, \dots ,Q \end{aligned}$$

12 $$\begin{aligned} {{\tilde{m}}_i}(t)&= {} m_{i}(t)- m_{i}(0) \quad \ i=1, \dots ,Q \end{aligned}$$

With the shifting of energy and magnetization, we can compute the mean of absolute energy per site $\mu _{|{\tilde{e}}(t)|}$, the mean of absolute magnetization per site $\mu _{|{\tilde{m}}(t)|}$, standard deviation of energy per site std$({\tilde{e}}(t))$, and standard deviation of magnetization per site std$({\tilde{m}}(t))$ over independent samples.

In Fig. 2, the spin-spin correlation plots are shown as using reference trajectory generated at the reference time step $\uptau =10^{-6}$ for ${k_B}T/J=0.4 \ ({k_B}T/J<{k_B}T_{c}/J)$ [Fig. 2a,d], ${k_B}T/J=1.44 \ ({k_B}T/J \approx {k_B}T_{c}/J)$ [Fig. 2b,e], and ${k_B}T/J=2.4 \ ({k_B}T/J>{k_B}T_{c}/J)$ [Fig. 2c,f]. At ${k_B}T/J<{k_B}T_{c}/J$ , correlations remain high (red line, yellow line, and blue line) except for at $\uptau =10^{-1}$ without Deep Learning corrections (black line), where correlation drops around $t=2$. This is due to accumulation of errors for large time steps. Correlation is recovered with Deep Learning corrections (blue line). Indeed correlations of $\uptau =10^{-1}$ with Deep Learning corrections are as good as for $\uptau =10^{-2}$ without Deep Learning corrections (yellow line), demonstrating a $~\sim 10$ times speed up. At ${k_B}T/J \approx {k_B}T_{c}/J$ and ${k_B}T/J>{k_B}T_{c}/J$ , spin-spin correlation drops faster than ${k_B}T/J<{k_B}T_{c}/J$ even for short time steps, $\uptau =10^{-4}$ (green line) and $\uptau =10^{-5}$ (violet line), due to disorder in the spin lattices.

We define threshold time $t_{thres}$ as the average time required for spin-spin correlation $\mu _{\xi (t)}$ to drop from 1 to 0.99. In Fig. 2g, the plot of $t_{thres}$ as a function of temperature ${k_B}T/J$ has the logarithmic scale on the *y*-axis, and simulations for $\uptau =10^{-3}$ have higher threshold time (red squares) at each temperature than for $\uptau =10^{-1}$ without Deep Learning corrections. Threshold time (filled blue diamonds) for $\uptau =10^{-1}$ with Deep Learning corrections approaches to almost the same threshold time (yellow circles) for $\uptau =10^{-2}$ without Deep Learning corrections at each temperature.

Figure 3 ($$L=4$$) and Fig. 4 ($$L=8$$) show $$\mu _{|{\tilde{e}}(t)|}$$, std$$({\tilde{e}}(t))$$, $$\mu _{|{\tilde{m}}(t)|}$$, and std$$({\tilde{m}}(t))$$ as a function of time at $${k_B}T/J=0.4$$$$({k_B}T/J<{k_B}T_{c}/J)$$ [Figs. 3a and 4a], $${k_B}T/J=1.44$$$$({k_B}T/J \approx {k_B}T_{c}/J)$$ [Figs. 3b and 4b], and $${k_B}T/J=2.4$$$$({k_B}T/J > {k_B}T_{c}/J)$$ [Figs. 3c and 4c]. For time steps $$\uptau =10^{-2}$$ (yellow line) and $$\uptau =10^{-3}$$ (red line), conservation of both energy and magnetization is good, as shown by the relatively constant mean plots ($$\mu _{|{\tilde{e}}(t)|}$$ and $$\mu _{|{\tilde{m}}(t)|}$$) and small standard deviations (std$$({\tilde{e}}(t))$$ and std$$({\tilde{m}}(t))$$) across independent simulations. At $${k_B}T/J < {k_B}T_{c}/J$$ and $${k_B}T/J \approx {k_B}T_{c}/J$$, both energy and magnetization are not conserved in simulations without Deep Learning corrections for time step $$\uptau =10^{-1}$$ (black line). On the other hand, conservation is recovered using Deep Learning corrections (blue line). In Fig. 3c, at $${k_B}T/J > {k_B}T_{c}/J$$, the system is disordered and the mean of absolute energy $$\mu _{|{\tilde{e}}(t)|}$$ and the mean of absolute magnetization $$\mu _{|{\tilde{m}}(t)|}$$ become more constant, simply due to averaging of disordered spins. Especially, Fig. 4c shows that at $${k_B}T/J > {k_B}T_{c}/J$$, the effect of averaging over disordered spins for $$L=8$$ is stronger than for $$L=4$$. At high temperature, the number of possible states increase exponentially and hence fitting by Deep Learning corrections is more difficult.

## Discussion

Our results have demonstrated that the Deep Learning corrections enhance the time integration step of the original Suzuki–Trotter method and have achieved $~\sim 10$ times computational speed up while maintaining accuracy compared to the original Suzuki–Trotter decomposition method. The nature of local nearest neighbours interactions in the lattice means that convolutional structure of the Deep Neural Network is a nature choice of network architecture. Since convolution is translationally invariant, the effect of lattice size on training our U-Net is not a major concern. For example, between $L=4$ and $L=8$ lattices, the time required for training the U-Net parameters increases by about 4 times, which is sub-linear with respect to the number of lattice sites. Our Deep Learning was trained on simulation data at $\uptau =10^{-3}$, however its accuracy performance is equivalent to simulation data at $\uptau =10^{-2}$. This shows that our Deep Learning training has not reached its theoretical limit of a perfect prediction. This theoretical limit can be achieved exactly if we train on an infinite amount of data for an infinite capacity. In practise, Deep Learning methods can not be perfect because the amount of data and the capacity of U-Net are finite. The main source of inaccuracies in our Deep Learning method is that U-Net’s output does not fit exactly the labeled data generated at $\uptau =10^{-3}$ and that even if U-Net is able to fit the data it has through training, it may not predict perfectly on the data it has never seen in training. For future work, we will explore the effects of Deep Learning corrections on higher order Suzuki–Trotter decomposition. We will also apply Deep Learning corrections such as off lattice systems and integrators such as velocity-verlet.

## Supplementary information

Supplementary Information.

## Data Availability

The data and code that support the findings of this study are available from corresponding authors upon request.
