# Supplementary material for: Accelerated spin dynamics using deep learning corrections
Source: Sci Rep. 2020 Aug 13;10:13772. doi: 10.1038/s41598-020-70558-1 (PMC7426868; doi:10.1038/s41598-020-70558-1)
Supplement: Supplementary file 1 — Supplementary Information. [file 41598_2020_70558_MOESM1_ESM.pdf]

Supplemental Information for Accelerated spin dynamics using Deep Learning corrections

Sojeong Park[1,2], Wooseop Kwak[1], and Hwee Kuan Lee[2,3,4,5,*]

[1]Department of Physics, Chosun University, Gwangju 61452, Republic of Korea
[2]Bioinformatics Institute, Agency for Science, Technology and Research (A*STAR), 30 Biopolis Street, #07-01 Matrix, 138671, Singapore, Singapore
[3]School of Computing, National University of Singapore, 13 Computing Drive, 117417, Singapore, Singapore
[4]Singapore Eye Research Institute (SERI), 11 Third Hospital Ave, 168751, Singapore, Singapore
[5]Image and Pervasive Access Laboratory (IPAL), 1 Fusionopolis Way, #21-01 Connexis (South Tower), 138632, Singapore, Singapore
[*]e-mail:leehk@bii.a-star.edu.sg

1 Details of the data preparation using simulated annealing method

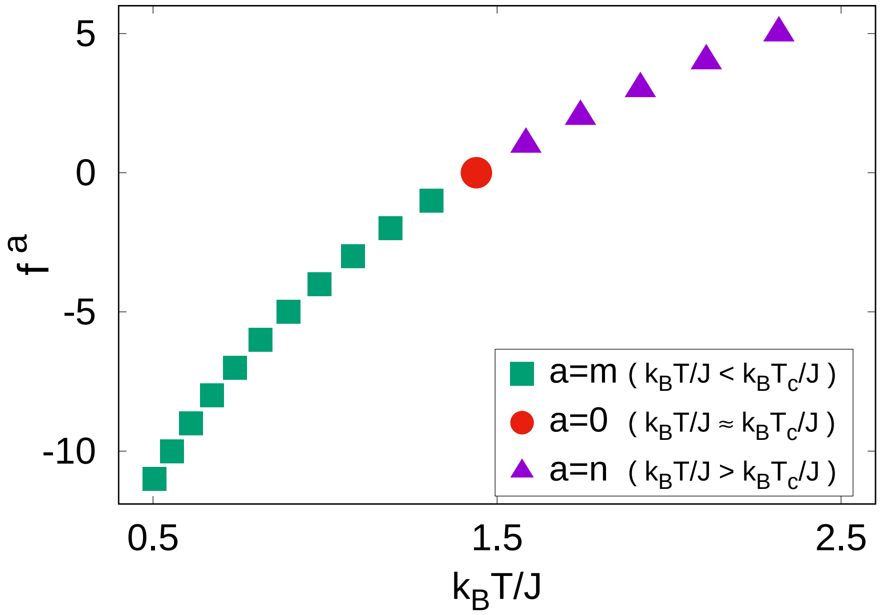

Figure S1. Plot of f^{a} as a function of temperature k_BT/J used for the simulated annealing methods, where f is modification factor and a is power of f.

In the Monte Carlo simulation, transition probability from one state to another state is $W = e^{-\beta\Delta E}$ for $\Delta E < 0$ in the metropolis algorithm. Since an inverse temperature β goes to infinite at low temperature, it is easy to fall into the local minima because the transition probability is $W = 0$. For the purpose of avoiding the local minima problem, we gradually lower the temperature from a high temperature to a low temperature using the simulated annealing method. Temperature dataset shown in figure S1 are generated in range of temperature $k_BT/J \in [0.5, 2.4]$, where k_BT/J is defined as follows:

$$k_BT/J = f^{-m}k_BT_c/J \quad , k_BT/J < k_BT_c/J \tag{1}$$

$$k_BT/J = f^{n}k_BT_c/J \quad , k_BT/J > k_BT_c/J \; . \tag{2}$$

Here, the positive real number f, the modification factor, is used to adjust the number of temperature dataset between minimum temperature $k_BT_{min}/J = 0.5$ and maximum temperature $k_BT_{max}/J = 2.4$, where m is the number of temperature dataset between the critical temperature k_BT_c/J and k_BT_{max}/J and n is the number of temperature dataset between k_BT_{min}/J and k_BT_c/J. The values of m and n are estimated as follows:

$$m = \frac{logk_BT_c/J - logk_BT/J}{logf} \tag{3}$$

$$n = \frac{logk_BT/J - logk_BT_c/J}{logf} \; , \tag{4}$$

where $k_BT_c/J \approx 1.44$, $m = 11$, $n = 5$, and $f = 1.1$ are used in this paper. The initial spin configurations are obtained below, near, and above the critical temperature. Below the critical temperature, 30,000 spin configurations representing order states are generated by using the Monte Carlo simulation at temperatures k_BT/J = 0.5, 0.56, 0.61, 0.67, 0.74, 0.81, 0.89, 0.98, 1.08, 1.19

each. Near the critical temperature, 70,000 spin configurations are generated at temperatures $k_B T/J$ = 1.31, 1.44, 1.58 each. Above the critical temperature, 100,000 spin configurations representing disordered states are generated at temperatures $k_B T/J$ = 1.74, 1.92, 2.11, 2.32 each.

Below the critical temperature, the entropy is low so the generated number of initial spin configurations are smaller than other temperatures. As the temperature increases as the entropy is high, we increase the number of initial spin configurations.

2 Simple U-Net architecture on lattice size $L = 4$

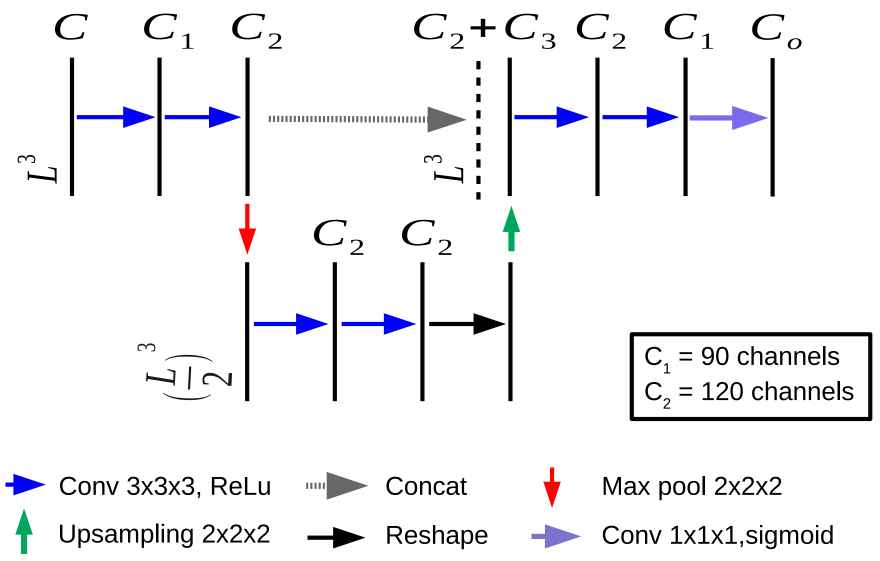

Figure S2. Illustration of the U-Net architecture. The architecture consists of encoder and decoder layers. Each vertical black line represents a multi-channel feature map. The number of channels is denoted on the top of straight vertical black line and each map's dimension is indicated on the left edge. Vertical dashed black lines correspond on the copied feature maps from each encoder layer.

The architecture of U-Net described in figure S2 is used for $4 \times 4 \times 4$ cubic lattice. Convolutional layers are used as an encoder followed by a decoder that consists of upsamplings and concatenations with the correspondingly feature maps from the encoder. The input dimensions of U-Net are reshaped to $[D, L, L, L, C]$ as cubic grid vector map. D is total number of training data and input channels C is 6 (3+3) by concatenating spin coordinates S_x, S_y, and S_z of σ_i and $\sigma_i^{(10^{-1})}$, respectively. The encoder consists of the repeated two convolutional layers with $3 \times 3 \times 3$ filters followed by a $2 \times 2 \times 2$ max pooling. Every step in decoder consists of upsampling layers with a $2 \times 2 \times 2$ filters followed by the repeated two convolutional layers with $3 \times 3 \times 3$ filters and copy with correspondingly cropped feature map from encoding layers. The periodic boundary conditions are also applied to the convolutional layers. The activation function of the output is a sigmoid for predicting values of residue with $[D, L, L, L, C_o]$ dimensions, where the number of output channels C_o is 3.
